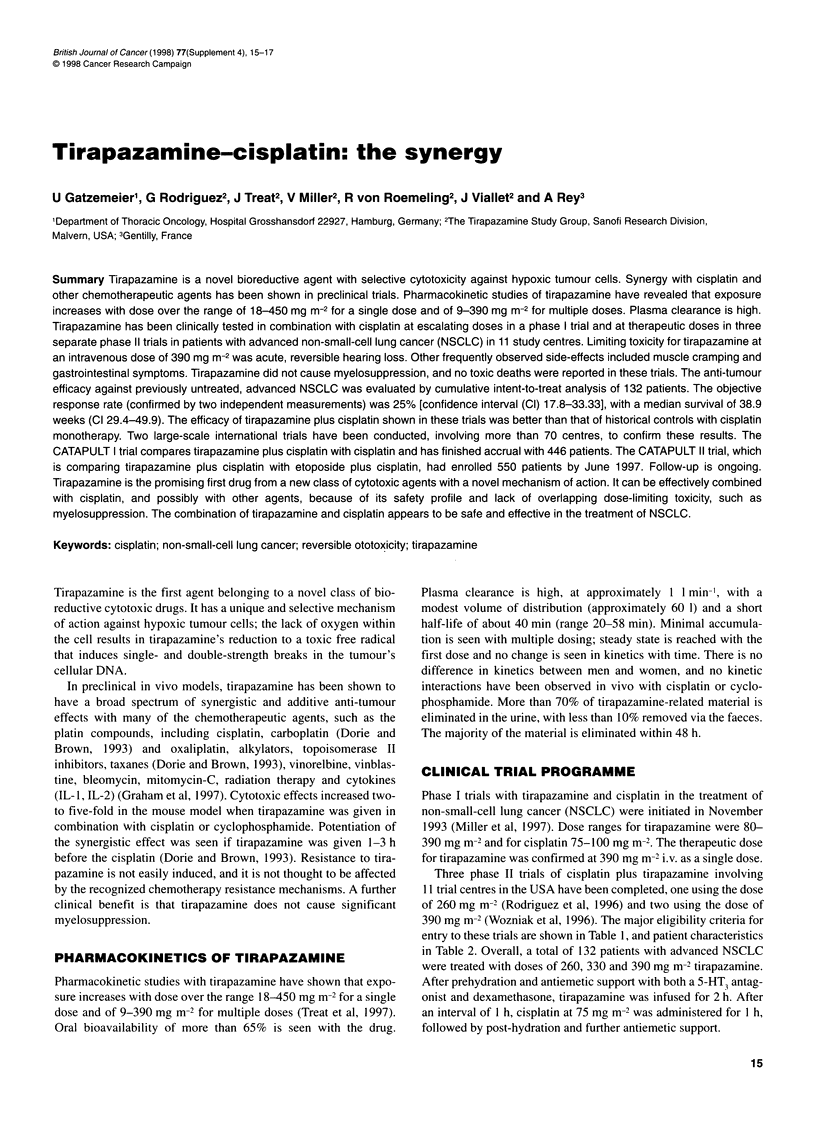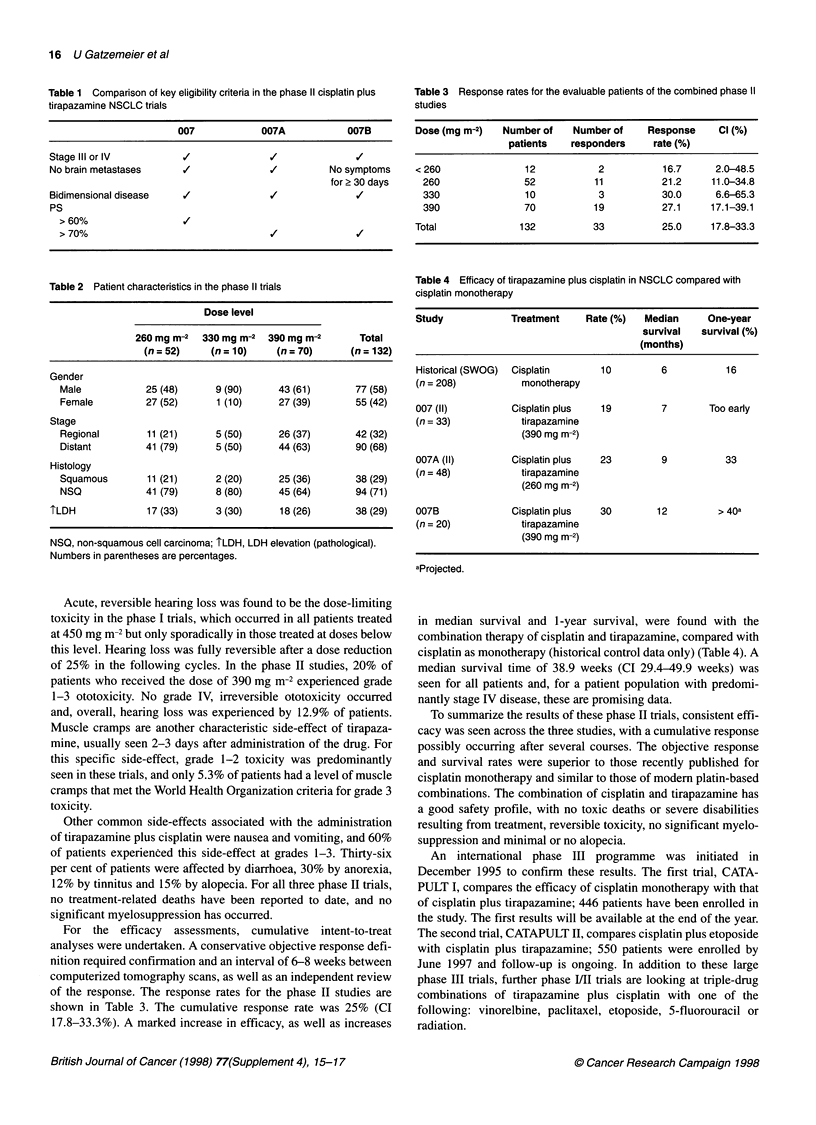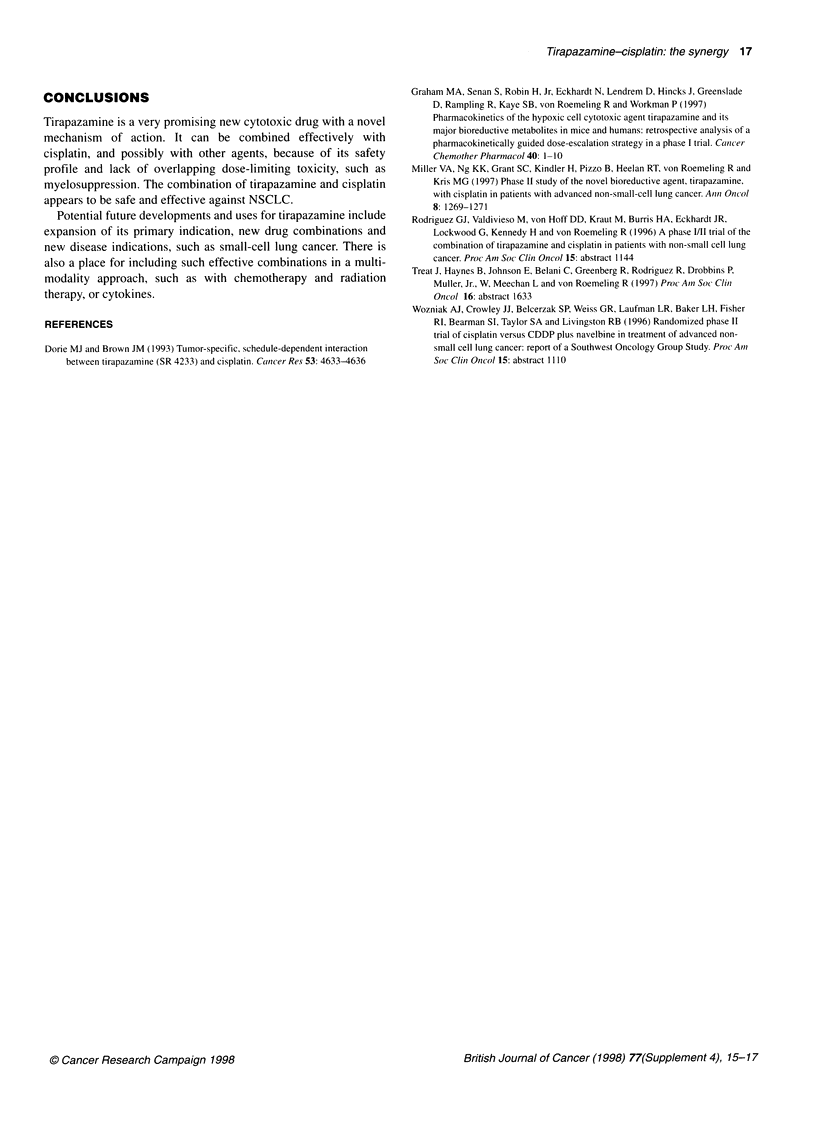# Tirapazamine-cisplatin: the synergy.

**DOI:** 10.1038/bjc.1998.431

**Published:** 1998-06

**Authors:** U. Gatzemeier, G. Rodriguez, J. Treat, V. Miller, R. von Roemeling, J. Viallet, A. Rey

**Affiliations:** Department of Thoracic Oncology, Hospital Grosshansdorf, Hamburg, Germany.

## Abstract

Tirapazamine is a novel bioreductive agent with selective cytotoxicity against hypoxic tumour cells. Synergy with cisplatin and other chemotherapeutic agents has been shown in preclinical trials. Pharmacokinetic studies of tirapazamine have revealed that exposure increases with dose over the range of 18-450 mg m(-2) for a single dose and of 9-390 mg m(-2) for multiple doses. Plasma clearance is high. Tirapazamine has been clinically tested in combination with cisplatin at escalating doses in a phase I trial and at therapeutic doses in three separate phase II trials in patients with advanced non-small-cell lung cancer (NSCLC) in 11 study centres. Limiting toxicity for tirapazamine at an intravenous dose of 390 mg m(-2) was acute, reversible hearing loss. Other frequently observed side-effects included muscle cramping and gastrointestinal symptoms. Tirapazamine did not cause myelosuppression, and no toxic deaths were reported in these trials. The anti-tumour efficacy against previously untreated, advanced NSCLC was evaluated by cumulative intent-to-treat analysis of 132 patients. The objective response rate (confirmed by two independent measurements) was 25% [confidence interval (CI) 17.8-33.33], with a median survival of 38.9 weeks (CI 29.4-49.9). The efficacy of tirapazamine plus cisplatin shown in these trials was better than that of historical controls with cisplatin monotherapy. Two large-scale international trials have been conducted, involving more than 70 centres, to confirm these results. The CATAPULT I trial compares tirapazamine plus cisplatin with cisplatin and has finished accrual with 446 patients. The CATAPULT II trial, which is comparing tirapazamine plus cisplatin with etoposide plus cisplatin, had enrolled 550 patients by June 1997. Follow-up is ongoing. Tirapazamine is the promising first drug from a new class of cytotoxic agents with a novel mechanism of action. It can be effectively combined with cisplatin, and possibly with other agents, because of its safety profile and lack of overlapping dose-limiting toxicity, such as myelosuppression. The combination of tirapazamine and cisplatin appears to be safe and effective in the treatment of NSCLC.


British Journal of Cancer (1998) 77(Supplement 4), 15–17
© 1998 Cancer Research Campaign

### Tirapazamine–cisplatin: the synergy

U Gatzemeier[1], G Rodriguez[2], J Treat[2], V Miller[2], R von Roemeling[2], J Viallet[2] and A Rey[3]

[1]Department of Thoracic Oncology, Hospital Grosshansdorf 22927, Hamburg, Germany; [2]The Tirapazamine Study Group, Sanofi Research Division, Malvern, USA; [3]Gentilly, France

**Summary** Tirapazamine is a novel bioreductive agent with selective cytotoxicity against hypoxic tumour cells. Synergy with cisplatin and other chemotherapeutic agents has been shown in preclinical trials. Pharmacokinetic studies of tirapazamine have revealed that exposure increases with dose over the range of 18–450 mg $m^{-2}$ for a single dose and of 9–390 mg $m^{-2}$ for multiple doses. Plasma clearance is high. Tirapazamine has been clinically tested in combination with cisplatin at escalating doses in a phase I trial and at therapeutic doses in three separate phase II trials in patients with advanced non-small-cell lung cancer (NSCLC) in 11 study centres. Limiting toxicity for tirapazamine at an intravenous dose of 390 mg $m^{-2}$ was acute, reversible hearing loss. Other frequently observed side-effects included muscle cramping and gastrointestinal symptoms. Tirapazamine did not cause myelosuppression, and no toxic deaths were reported in these trials. The anti-tumour efficacy against previously untreated, advanced NSCLC was evaluated by cumulative intent-to-treat analysis of 132 patients. The objective response rate (confirmed by two independent measurements) was 25% [confidence interval (CI) 17.8–33.33], with a median survival of 38.9 weeks (CI 29.4–49.9). The efficacy of tirapazamine plus cisplatin shown in these trials was better than that of historical controls with cisplatin monotherapy. Two large-scale international trials have been conducted, involving more than 70 centres, to confirm these results. The CATAPULT I trial compares tirapazamine plus cisplatin with cisplatin and has finished accrual with 446 patients. The CATAPULT II trial, which is comparing tirapazamine plus cisplatin with etoposide plus cisplatin, had enrolled 550 patients by June 1997. Follow-up is ongoing. Tirapazamine is the promising first drug from a new class of cytotoxic agents with a novel mechanism of action. It can be effectively combined with cisplatin, and possibly with other agents, because of its safety profile and lack of overlapping dose-limiting toxicity, such as myelosuppression. The combination of tirapazamine and cisplatin appears to be safe and effective in the treatment of NSCLC.

**Keywords:** cisplatin; non-small-cell lung cancer; reversible ototoxicity; tirapazamine

Tirapazamine is the first agent belonging to a novel class of bioreductive cytotoxic drugs. It has a unique and selective mechanism of action against hypoxic tumour cells; the lack of oxygen within the cell results in tirapazamine's reduction to a toxic free radical that induces single- and double-strength breaks in the tumour's cellular DNA.

In preclinical in vivo models, tirapazamine has been shown to have a broad spectrum of synergistic and additive anti-tumour effects with many of the chemotherapeutic agents, such as the platin compounds, including cisplatin, carboplatin (Dorie and Brown, 1993) and oxaliplatin, alkylators, topoisomerase II inhibitors, taxanes (Dorie and Brown, 1993), vinorelbine, vinblastine, bleomycin, mitomycin-C, radiation therapy and cytokines (IL-1, IL-2) (Graham et al, 1997). Cytotoxic effects increased two- to five-fold in the mouse model when tirapazamine was given in combination with cisplatin or cyclophosphamide. Potentiation of the synergistic effect was seen if tirapazamine was given 1–3 h before the cisplatin (Dorie and Brown, 1993). Resistance to tirapazamine is not easily induced, and it is not thought to be affected by the recognized chemotherapy resistance mechanisms. A further clinical benefit is that tirapazamine does not cause significant myelosuppression.

### PHARMACOKINETICS OF TIRAPAZAMINE

Pharmacokinetic studies with tirapazamine have shown that exposure increases with dose over the range 18–450 mg $m^{-2}$ for a single dose and of 9–390 mg $m^{-2}$ for multiple doses (Treat et al, 1997). Oral bioavailability of more than 65% is seen with the drug. Plasma clearance is high, at approximately 1 l $min^{-1}$, with a modest volume of distribution (approximately 60 l) and a short half-life of about 40 min (range 20–58 min). Minimal accumulation is seen with multiple dosing; steady state is reached with the first dose and no change is seen in kinetics with time. There is no difference in kinetics between men and women, and no kinetic interactions have been observed in vivo with cisplatin or cyclophosphamide. More than 70% of tirapazamine-related material is eliminated in the urine, with less than 10% removed via the faeces. The majority of the material is eliminated within 48 h.

### CLINICAL TRIAL PROGRAMME

Phase I trials with tirapazamine and cisplatin in the treatment of non-small-cell lung cancer (NSCLC) were initiated in November 1993 (Miller et al, 1997). Dose ranges for tirapazamine were 80–390 mg $m^{-2}$ and for cisplatin 75–100 mg $m^{-2}$. The therapeutic dose for tirapazamine was confirmed at 390 mg $m^{-2}$ i.v. as a single dose.

Three phase II trials of cisplatin plus tirapazamine involving 11 trial centres in the USA have been completed, one using the dose of 260 mg $m^{-2}$ (Rodriguez et al, 1996) and two using the dose of 390 mg $m^{-2}$ (Wozniak et al, 1996). The major eligibility criteria for entry to these trials are shown in Table 1, and patient characteristics in Table 2. Overall, a total of 132 patients with advanced NSCLC were treated with doses of 260, 330 and 390 mg $m^{-2}$ tirapazamine. After prehydration and antiemetic support with both a 5-$HT_3$ antagonist and dexamethasone, tirapazamine was infused for 2 h. After an interval of 1 h, cisplatin at 75 mg $m^{-2}$ was administered for 1 h, followed by post-hydration and further antiemetic support.

**Table 1** Comparison of key eligibility criteria in the phase II cisplatin plus tirapazamine NSCLC trials

| | 007 | 007A | 007B |
|---|---|---|---|
| Stage III or IV | ✓ | ✓ | ✓ |
| No brain metastases | ✓ | ✓ | No symptoms for ≥ 30 days |
| Bidimensional disease | ✓ | ✓ | ✓ |
| PS | | | |
| > 60% | ✓ | | |
| > 70% | | ✓ | ✓ |

**Table 2** Patient characteristics in the phase II trials

| | Dose level | | | |
|---|---|---|---|---|
| | 260 mg m$^{-2}$ (*n* = 52) | 330 mg m$^{-2}$ (*n* = 10) | 390 mg m$^{-2}$ (*n* = 70) | Total (*n* = 132) |
| Gender | | | | |
| Male | 25 (48) | 9 (90) | 43 (61) | 77 (58) |
| Female | 27 (52) | 1 (10) | 27 (39) | 55 (42) |
| Stage | | | | |
| Regional | 11 (21) | 5 (50) | 26 (37) | 42 (32) |
| Distant | 41 (79) | 5 (50) | 44 (63) | 90 (68) |
| Histology | | | | |
| Squamous | 11 (21) | 2 (20) | 25 (36) | 38 (29) |
| NSQ | 41 (79) | 8 (80) | 45 (64) | 94 (71) |
| ↑LDH | 17 (33) | 3 (30) | 18 (26) | 38 (29) |

NSQ, non-squamous cell carcinoma; ↑LDH, LDH elevation (pathological). Numbers in parentheses are percentages.

**Table 3** Response rates for the evaluable patients of the combined phase II studies

| Dose (mg m$^{-2}$) | Number of patients | Number of responders | Response rate (%) | CI (%) |
|---|---|---|---|---|
| < 260 | 12 | 2 | 16.7 | 2.0–48.5 |
| 260 | 52 | 11 | 21.2 | 11.0–34.8 |
| 330 | 10 | 3 | 30.0 | 6.6–65.3 |
| 390 | 70 | 19 | 27.1 | 17.1–39.1 |
| Total | 132 | 33 | 25.0 | 17.8–33.3 |

**Table 4** Efficacy of tirapazamine plus cisplatin in NSCLC compared with cisplatin monotherapy

| Study | Treatment | Rate (%) | Median survival (months) | One-year survival (%) |
|---|---|---|---|---|
| Historical (SWOG) (*n* = 208) | Cisplatin monotherapy | 10 | 6 | 16 |
| 007 (II) (*n* = 33) | Cisplatin plus tirapazamine (390 mg m$^{-2}$) | 19 | 7 | Too early |
| 007A (II) (*n* = 48) | Cisplatin plus tirapazamine (260 mg m$^{-2}$) | 23 | 9 | 33 |
| 007B (*n* = 20) | Cisplatin plus tirapazamine (390 mg m$^{-2}$) | 30 | 12 | > 40[a] |

[a]Projected.

Acute, reversible hearing loss was found to be the dose-limiting toxicity in the phase I trials, which occurred in all patients treated at 450 mg m$^{-2}$ but only sporadically in those treated at doses below this level. Hearing loss was fully reversible after a dose reduction of 25% in the following cycles. In the phase II studies, 20% of patients who received the dose of 390 mg m$^{-2}$ experienced grade 1–3 ototoxicity. No grade IV, irreversible ototoxicity occurred and, overall, hearing loss was experienced by 12.9% of patients. Muscle cramps are another characteristic side-effect of tirapazamine, usually seen 2–3 days after administration of the drug. For this specific side-effect, grade 1–2 toxicity was predominantly seen in these trials, and only 5.3% of patients had a level of muscle cramps that met the World Health Organization criteria for grade 3 toxicity.

Other common side-effects associated with the administration of tirapazamine plus cisplatin were nausea and vomiting, and 60% of patients experienced this side-effect at grades 1–3. Thirty-six per cent of patients were affected by diarrhoea, 30% by anorexia, 12% by tinnitus and 15% by alopecia. For all three phase II trials, no treatment-related deaths have been reported to date, and no significant myelosuppression has occurred.

For the efficacy assessments, cumulative intent-to-treat analyses were undertaken. A conservative objective response definition required confirmation and an interval of 6–8 weeks between computerized tomography scans, as well as an independent review of the response. The response rates for the phase II studies are shown in Table 3. The cumulative response rate was 25% (CI 17.8–33.3%). A marked increase in efficacy, as well as increases in median survival and 1-year survival, were found with the combination therapy of cisplatin and tirapazamine, compared with cisplatin as monotherapy (historical control data only) (Table 4). A median survival time of 38.9 weeks (CI 29.4–49.9 weeks) was seen for all patients and, for a patient population with predominantly stage IV disease, these are promising data.

To summarize the results of these phase II trials, consistent efficacy was seen across the three studies, with a cumulative response possibly occurring after several courses. The objective response and survival rates were superior to those recently published for cisplatin monotherapy and similar to those of modern platin-based combinations. The combination of cisplatin and tirapazamine has a good safety profile, with no toxic deaths or severe disabilities resulting from treatment, reversible toxicity, no significant myelosuppression and minimal or no alopecia.

An international phase III programme was initiated in December 1995 to confirm these results. The first trial, CATAPULT I, compares the efficacy of cisplatin monotherapy with that of cisplatin plus tirapazamine; 446 patients have been enrolled in the study. The first results will be available at the end of the year. The second trial, CATAPULT II, compares cisplatin plus etoposide with cisplatin plus tirapazamine; 550 patients were enrolled by June 1997 and follow-up is ongoing. In addition to these large phase III trials, further phase I/II trials are looking at triple-drug combinations of tirapazamine plus cisplatin with one of the following: vinorelbine, paclitaxel, etoposide, 5-fluorouracil or radiation.

© Cancer Research Campaign 1998

### CONCLUSIONS

Tirapazamine is a very promising new cytotoxic drug with a novel mechanism of action. It can be combined effectively with cisplatin, and possibly with other agents, because of its safety profile and lack of overlapping dose-limiting toxicity, such as myelosuppression. The combination of tirapazamine and cisplatin appears to be safe and effective against NSCLC.

Potential future developments and uses for tirapazamine include expansion of its primary indication, new drug combinations and new disease indications, such as small-cell lung cancer. There is also a place for including such effective combinations in a multi-modality approach, such as with chemotherapy and radiation therapy, or cytokines.

### REFERENCES

Dorie MJ and Brown JM (1993) Tumor-specific, schedule-dependent interaction between tirapazamine (SR 4233) and cisplatin. *Cancer Res* **53**: 4633–4636

Graham MA, Senan S, Robin H, Jr, Eckhardt N, Lendrem D, Hincks J, Greenslade D, Rampling R, Kaye SB, von Roemeling R and Workman P (1997) Pharmacokinetics of the hypoxic cell cytotoxic agent tirapazamine and its major bioreductive metabolites in mice and humans: retrospective analysis of a pharmacokinetically guided dose-escalation strategy in a phase I trial. *Cancer Chemother Pharmacol* **40**: 1–10

Miller VA, Ng KK, Grant SC, Kindler H, Pizzo B, Heelan RT, von Roemeling R and Kris MG (1997) Phase II study of the novel bioreductive agent, tirapazamine, with cisplatin in patients with advanced non-small-cell lung cancer. *Ann Oncol* **8**: 1269–1271

Rodriguez GJ, Valdivieso M, von Hoff DD, Kraut M, Burris HA, Eckhardt JR, Lockwood G, Kennedy H and von Roemeling R (1996) A phase I/II trial of the combination of tirapazamine and cisplatin in patients with non-small cell lung cancer. *Proc Am Soc Clin Oncol* **15**: abstract 1144

Treat J, Haynes B, Johnson E, Belani C, Greenberg R, Rodriguez R, Drobbins P, Muller, Jr., W, Meechan L and von Roemeling R (1997) *Proc Am Soc Clin Oncol* **16**: abstract 1633

Wozniak AJ, Crowley JJ, Belcerzak SP, Weiss GR, Laufman LR, Baker LH, Fisher RI, Bearman SI, Taylor SA and Livingston RB (1996) Randomized phase II trial of cisplatin versus CDDP plus navelbine in treatment of advanced non-small cell lung cancer: report of a Southwest Oncology Group Study. *Proc Am Soc Clin Oncol* **15**: abstract 1110

© Cancer Research Campaign 1998